# Supplementary material for: One-minute sit-to-stand test: Reference values for the Chilean population
Source: PLoS One. 2025 Jan 29;20(1):e0317594. doi: 10.1371/journal.pone.0317594 (PMC11778653; doi:10.1371/journal.pone.0317594)

PLOS ONE

One-minute sit-to-stand test: Reference values for Chilean population.

--Manuscript Draft--

**Manuscript Number:**	PONE-D-24-27131
**Article Type:**	Research Article
**Full Title:**	One-minute sit-to-stand test: Reference values for Chilean population.
**Short Title:**	One-minute sit-to-stand test from Chile.
**Corresponding Author:**	Rodrigo Torres-Castro<br>Universidad de Chile<br>Santiago, Santiago CHILE
**Keywords:**	Functional capacity, field tests, Reference values, Sit-to-stand test, Physical Performance.
**Abstract:**	Introduction: The one-minute Sit and Stand Test (1min-STST) is a field test used to assess functional capacity. It is easily implementable and of significant clinical utility; however, no reference values are currently available for the Chilean population. The objective of this study was to establish reference values for the 1min-STST from a healthy Chilean population.<br>Methods: A multicentre cross-sectional study was conducted, involving data collection from six locations in Chile. Healthy adults between 18 and 80 were recruited. The anthropometric variables, levels of physical activity, smoking status, Borg scale ratings, and the number of repetitions during the 1min-STST were recorded. Reference values were determined according to sex and age range.<br>Results: Four hundred and ninety-nine healthy subjects (57.5% women, n=287, median of height of 1.63 (0.14) m, a weight of 72.8(20) kg, and an average of BMI of 27.3 ± 4.1 kg/m2) were included in the study. . The median (and the lower limit of normality values) for the 1min-STST in men ranged from 18-29 years was 38 (LLN 27) repetitions and 23 (LLN 15) repetitions for 70-80 years. For women aged 18-29 years there were 38 (LLN 28) repetitions and for range between 70-80 years there were 24 (LLN 17).<br>Conclusions: This study established reference values for the healthy adult Chilean population.
**Order of Authors:**	Matías Otto-Yáñez, PhD
	Rodrigo Torres-Castro
	Marisol Barros-Poblete
	Marcela Barros
	Carola Valencia
	Alex Campos
	Leticia Jadue
	Homero Puppo
	Pamela Serón
	Jordi Vilaró
**Additional Information:**	
**Question**	**Response**
**Financial Disclosure**<br><br>Enter a financial disclosure statement that describes the sources of funding for the work included in this submission. Review the submission guidelines for detailed	The author(s) received no specific funding for this work.

Powered by Editorial Manager® and ProduXion Manager® from Aries Systems Corporation

requirements. View published research articles from *PLOS ONE* for specific examples.<br><br>This statement is required for submission and **will appear in the published article** if the submission is accepted. Please make sure it is accurate.<br><br>**Funded studies**<br>Enter a statement with the following details:<br>• Initials of the authors who received each award<br>• Grant numbers awarded to each author<br>• The full name of each funder<br>• URL of each funder website<br>• Did the sponsors or funders play any role in the study design, data collection and analysis, decision to publish, or preparation of the manuscript?<br><br>Did you receive funding for this work?	
**Competing Interests**<br><br>Use the instructions below to enter a competing interest statement for this submission. On behalf of all authors, disclose any competing interests that could be perceived to bias this work—acknowledging all financial support and any other relevant financial or non-financial competing interests.<br><br>This statement is required for submission and **will appear in the published article** if the submission is accepted. Please make sure it is accurate and that any funding sources listed in your Funding Information later in the submission form are also declared in your Financial Disclosure statement.<br><br>View published research articles from *PLOS ONE* for specific examples.	None of the authors declare any conflict of interest for this research

**NO authors have competing interests**<br><br>Enter: *The authors have declared that no competing interests exist.*<br><br>**Authors with competing interests**<br><br>Enter competing interest details beginning with this statement:<br><br>*I have read the journal's policy and the authors of this manuscript have the following competing interests: [insert competing interests here]*<br><br>* typeset	
**Ethics Statement**<br><br>Enter an ethics statement for this submission. This statement is required if the study involved:<br><br>• Human participants<br>• Human specimens or tissue<br>• Vertebrate animals or cephalopods<br>• Vertebrate embryos or tissues<br>• Field research<br><br>Write "N/A" if the submission does not require an ethics statement.<br><br>General guidance is provided below. Consult the submission guidelines for detailed instructions. **Make sure that all information entered here is included in the Methods section of the manuscript.**	This study was approved by the Ethics Committee of the Metropolitan Eastern Health Service (14-06-2019). All subjects gave written consent.

**Format for specific study types**<br><br>**Human Subject Research (involving human participants and/or tissue)**<br>• Give the name of the institutional review board or ethics committee that approved the study<br>• Include the approval number and/or a statement indicating approval of this research<br>• Indicate the form of consent obtained (written/oral) or the reason that consent was not obtained (e.g. the data were analyzed anonymously)<br><br>**Animal Research (involving vertebrate animals, embryos or tissues)**<br>• Provide the name of the Institutional Animal Care and Use Committee (IACUC) or other relevant ethics board that reviewed the study protocol, and indicate whether they approved this research or granted a formal waiver of ethical approval<br>• Include an approval number if one was obtained<br>• If the study involved *non-human primates*, add *additional details* about animal welfare and steps taken to ameliorate suffering<br>• If anesthesia, euthanasia, or any kind of animal sacrifice is part of the study, include briefly which substances and/or methods were applied<br><br>**Field Research**<br><br>Include the following details if this study involves the collection of plant, animal, or other materials from a natural setting:<br>• Field permit number<br>• Name of the institution or relevant body that granted permission	
**Data Availability**<br><br>Authors are required to make all data underlying the findings described fully available, without restriction, and from the time of publication. PLOS allows rare exceptions to address legal and ethical concerns. See the PLOS Data Policy and FAQ for detailed information.	Yes - all data are fully available without restriction

Powered by Editorial Manager® and ProduXion Manager® from Aries Systems Corporation

A Data Availability Statement describing where the data can be found is required at submission. Your answers to this question constitute the Data Availability Statement and **will be published in the article**, if accepted.<br><br>**Important:** Stating 'data available on request from the author' is not sufficient. If your data are only available upon request, select 'No' for the first question and explain your exceptional situation in the text box.<br><br>Do the authors confirm that all data underlying the findings described in their manuscript are fully available without restriction?	
**Describe where the data may be found in full sentences. If you are copying our sample text, replace any instances of XXX with the appropriate details.**<br><br>• If the data are **held or will be held in a public repository**, include URLs, accession numbers or DOIs. If this information will only be available after acceptance, indicate this by ticking the box below. For example: *All XXX files are available from the XXX database (accession number(s) XXX, XXX.)*.<br>• If the data are all contained **within the manuscript and/or Supporting Information files**, enter the following: *All relevant data are within the manuscript and its Supporting Information files.*<br>• If neither of these applies but you are able to provide **details of access elsewhere**, with or without limitations, please do so. For example:<br><br>*Data cannot be shared publicly because of [XXX]. Data are available from the XXX Institutional Data Access / Ethics Committee (contact via XXX) for researchers who meet the criteria for access to confidential data.*<br><br>*The data underlying the results presented in the study are available from (include the name of the third party*	All relevant data are within the manuscript and its Supporting Information files.

and contact information or URL).<br>• This text is appropriate if the data are owned by a third party and authors do not have permission to share the data.<br><br>* typeset	
Additional data availability information:	

Manuscrito
Click here to access/download;Manuscript;paper 1minsts reference values July 2.docx

One-minute sit-to-stand test: Reference values for Chilean population.

Running title: One-minute sit-to-stand test from Chile.

Matías Otto-Yáñez[1], Rodrigo Torres Castro[2*], Marisol Barros-Poblete[3], Marcela Barros[4], Carola Valencia[5], Alex Campos[6], Leticia Jadue[7], Homero Puppo[2], Pamela Seron[8], Jordi Vilaró[9]

Affiliations: [1]Grupo de Investigación en Salud, Funcionalidad y Actividad Física (GISFAF), Kinesiología, Facultad de Ciencias de la Salud, Universidad Autónoma de Chile, Santiago, Chile; [2]Department of Physical Therapy, Faculty of Medicine, University of Chile, Santiago, Chile; [3]Programa Doctorado en Ciencias Médicas, Universidad Austral de Chile, Valdivia, Chile; [4]CESFAM Eduardo Frei, Villa Alemana, Chile; [5]Centro de salud Dr Miguel Concha, Quillota, Chile; [6]CESFAM Alberto Allende Jones, Talagante, Chile; [7]Escuela de Kinesiología, Universidad de Santiago, Santiago, Chile; [8]Universidad de La Frontera, Departamento de Ciencias de la Rehabilitación, Temuco, Chile; [9]Blanquerna School of Health Sciences, Global Research on Wellbeing (GRoW), Ramon Llull University, Barcelona, Spain.

Address for correspondence: Rodrigo Torres-Castro. Department of Physical Therapy, University of Chile, Santiago, Chile. Phone: +56229786513. E-mail: rodritorres@uchile.cl

Conflict of Interest: None

Funding: No funding was received for this research.

ABSTRACT

Introduction: The one-minute Sit and Stand Test (1min-STST) is a field test used to assess functional capacity. It is easily implementable and of significant clinical utility; however, no reference values are currently available for the Chilean population. The objective of this study was to establish reference values for the 1min-STST from a healthy Chilean population.

Methods: A multicentre cross-sectional study was conducted, involving data collection from six locations in Chile. Healthy adults between 18 and 80 were recruited. The anthropometric variables, levels of physical activity, smoking status, Borg scale ratings, and the number of repetitions during the 1min-STST were recorded. Reference values were determined according to sex and age range.

Results: Four hundred and ninety-nine healthy subjects (57.5% women, n=287, median of height of 1.63 (0.14) m, a weight of 72.8(20) kg, and an average of BMI of 27.3 ± 4.1 kg/m^2) were included in the study. . The median (and the lower limit of normality values) for the 1min-STST in men ranged from 18-29 years was 38 (LLN 27) repetitions and 23 (LLN 15) repetitions for 70-80 years. For women aged 18-29 years there were 38 (LLN 28) repetitions and for range between 70-80 years there were 24 (LLN 17).

Conclusions: This study established reference values for the healthy adult Chilean population.

Keywords: Functional capacity, field tests, Reference values, Sit-to-stand test, Physical Performance.

Abbreviations

1min-STST: one-minute sit-to-stand test

BMI: Body mass index

IPAQ: International Physical Activity Questionnaire

LLN: Lower limit of normality

ULN: Upper limit of normality

Introduction

In a broad sense, functional capacity is the ability to carry out everyday tasks at home, school or work with enough energy to enjoy leisure-time activities and meet periods of unusual strain or disease [1]. It encompasses various aspects such as strength, endurance, flexibility, and speed, all of which influence one's physical performance and overall health [2]. This capacity is fundamental for daily tasks and participating in sports or recreational activities [2].

The evaluation of functional capacity is a crucial process for understanding an individual's level of physical fitness [3]. It can be conducted in a laboratory setting and through field tests [4]. Both methods have advantages and disadvantages; for example, the laboratory provides more precise and controlled measurements, such as gas and lactate analysis, while field tests are more practical and accessible for clinicians, measuring performance in more daily situations [5]. Field tests hold significant value, particularly when resources for laboratory evaluations are limited [6]. They offer a reasonable estimation of functional capacity without requiring expensive or personalised equipment [6]. Additionally, they are better suited for broader populations, such as areas with limited access to laboratory facilities, providing an overall view of the physical status of the population [7].

The 6-minute walk test (6MWT) has been widely used as a standard to assess functional capacity in various population groups due to its simplicity and effectiveness [6]. However, in situations where the 6MWT cannot be performed,

for example, due to lack of a corridor with adequate length, a viable alternative is the 1-minute sit-to-stand test (1min-STST) [8]. The 1min-STST is particularly valuable in assessing and monitoring patients with respiratory or cardiovascular conditions, providing insights into their functional capacity and the effectiveness of implemented treatments and therapies [9, 10].

The literature provides considerable support for the psychometric properties of using the 1min-STST to quantify functional capacity [11, 12]. The psychometric properties of the 1min-STST and other field tests are essential to ensure their validity and reliability. These properties refer to the test's ability to accurately and consistently measure an individual's functional capacity [13].

Because of this, 1min-STST has gained ground in rehabilitation by being a useful and easily accessible tool for monitoring patients in primary health care and telerehabilitation, facilitating access to and monitoring patients with physical limitations [14, 15]. Through these modalities, field tests like the 1min-STST can be administered more conveniently and accessibly, enabling remote assessment and tracking of patients' progress over time [16]. This benefits both healthcare professionals and patients, facilitating rehabilitation and improving care quality [16, 17].

When using field tests like the 1min-STST, having reference values is essential to interpret the obtained results in the appropriate context [18]. These reference values provide points of comparison to determine whether an individual's

performance falls within normal ranges for their age group and specific conditions [18]. Currently, the most used reference values are from Strassman et al. who offer a compilation of normative data that can serve as a reference for interpreting results in different populations and clinical settings [19]. However, these values were determined in a population with less sedentary lifestyle and obesity than the Latin American population, overestimating the expected performance for the subjects of our population [20].

While existing reference values can serve as a starting point, it is crucial to consider the variability among local populations and contexts. Factors such as age, sex, physical activity level and specific health conditions can influence test results [18]. There are ethnic, anthropometric, sociocultural, and dietary reasons that can influence the differences in the results of functional capacity tests in different populations, which would be another reason to justify having our own reference values [21]. Therefore, establishing local reference values and objectives will allow for a more precise and personalised evaluation of functional capacity and patient progress in their specific environment. We aimed to determine 1min-STST reference values from an adult healthy Chilean population between 18 to 80 years. Additionally, we compared the reference values with previous data and analysed by physical activity level. Our hypothesis is that the reference values for the 1-minute sit-to-stand test (1min-STS) in the Chilean adult population are different from those commonly used and correlate with age, sex, and physical activity level.

Methods

Study design and participants

A cross-sectional study was carried out simultaneously in six centres, three of these were universities and the other three were primary care health centres, across different geographical regions of Chile (Villa Alemana, Quilpué, Talagante, Santiago, Talca, Puerto Montt) from June 2019 to June 2023. This study was approved by the Ethics Committee of the Metropolitan Eastern Health Service (14-06-2019). All subjects gave written consent. This study was performed following the "Strengthening the Reporting of Observational Studies in Epidemiology" (STROBE) Guidelines [22].

Participants were recruited from the general population. The recruitment strategy was uniform across all centres and involved the dissemination of information through posters on the recruiters' social media platforms, physical posters placed both inside and outside the evaluation centres, and email outreach for individuals who voluntarily expressed interest in participating in the research and who met the inclusion criteria, which were: Adults between 18 and 80 years of age self-reported as healthy, who stated that they were able to stand up and sit down in a chair. Exclusion criteria: A BMI ≥ 35, have a chronic or acute respiratory disease in the last 30 days, have an acute or chronic musculoskeletal injury, concomitant cardiac, cerebral, or neuromuscular disease that prevents them from performing the tests and/or presents an inability to understand instructions.

Measurements

Each participant was assessed in one single visit using a standardised assessment order. Firstly, anthropometric and demographic characteristics were obtained. For smoking status, participants indicated if they were a “never smoker”, “ex- smoker” or “current smoker”. The abbreviated version of the International Physical Activity Questionnaire (IPAQ) was used to assess the physical activity level [23]. It was classified as low, moderate, and high level.

The 1min-STST test consisted of a simple movement: standing up from a chair, adopting the bipedal position with their knees in maximum extension [24]. A standard 43-46 cm chair was used, with thoracolumbar support [25]. Subjects sat upright on the chair against the wall with the knees and hips flexed and the feet flat on the floor at shoulder-width apart. The test's previous instruction stipulates that the subject has to complete the maximum feasible number of repetitions within one minute. The number of times fully seated and standing up in the chair during one minute was recorded as the primary variable and only one 1min-STS measurement was performed [26].. In addition, both at the beginning and end of the test, the perception of effort was evaluated through the modified Borg scale [27].

To standardise the measurements, training sessions were conducted, during which all evaluators were instructed to record three videos demonstrating the execution of the 1min-STST using a designated pilot test individual. The purpose was to ascertain adherence to the study protocol. Once the validation of the three

recorded evaluations was confirmed, authorisation was granted to start the study measurements.

Statistical analysis

Data were analysed using the statistical IBM SPSS version 25.0 (IBM Corporation, Armonk, NY, USA). The Kolmogorov Smirnov test was used to verify data distribution and numerical variables were presented as mean and standard deviation (SD), and qualitative variables were presented as frequency and percentage. A correlation analysis was also performed using the Pearson's or Spearman's test for the quantitative variables (age, weight, height, BMI, initial Borg, and final Borg) with the 1min-STST results. To establish the reference values, we used the categories already used previously [28]. Sex- and age-specific normative percentiles (2.5rd, 25th, 50th, 75th, and 97.5th) were generated.

To examine the relationship between individual performances in the 1min-STST test and the age of the subjects, we constructed dispersion graphs depicting the performance distribution concerning age and sex. To assess the impact of physical activity on 1min-STST performance, we compared the number of repetitions at different levels of physical activity, as measured by IPAQ-SF (low, medium, and high). This comparison was conducted using t-tests or Kruskal-Wallis tests.

Results of reference values are presented separately by sex and age groups. The proposed values by Strassmann (p50) were compared with those obtained from our study population. The comparison was made between the measured values and the 50th percentile (p50) reported in the Strassmann et al. study, according to sex and age range. In addition, a linear regression of these data was performed. To assess the differences between the two sets of results, a comparison was conducted using the student’s t-test. This statistical analysis aimed to determine whether there were significant discrepancies between the two datasets.

To calculate the sample size based on total population, we used a free University of Granada software. With a risk of type 1 error of 5% and confidence level of 97%. As a result, the final estimate determined a minimum of 471 participants.

To facilitate the calculation of reference values, multiple linear regression was performed for men and women, with performance in the 1min-STST as the dependent variable and age, height, and weight as predictors. The stepwise method was used for generating the predictive model.

Results

506 participants were recruited for the study. Seven subjects were excluded for having a BMI >35 kg/m². Therefore, the results of 499 people were analyzed,

comprising 287 women (57.5%) and 212 men (42.5%). Among the participants, 72.1% reported having a low level of physical activity, and 25.3% declared themselves smokers. Further information on the population's characteristics can be found in Table 1.

The correlations between the values of 1min-STST and age revealed a moderated inverse relationship ($r=-0.529$; $p<0.001$) a low correlation with Borg pre ($r=-0.253$; $p<0.001$), and Borg post ($r=0.286$; $p<0.001$), a very low correlation with the weight ($r=-0.120$; $p=0.006$) and a very weak correlation with BMI ($r=-0.109$; $p=0.015$). There was no correlation with height ($r=-0.019$; $p=0.7$).

The relationship between individual performances in the 1min-STST test and the age of the subjects is presented in Figure 1. These graphs visually represent how the 1min-STST performance varies across different age groups and between male and female participants.

Table 2 shows the results stratified according to percentiles for different age and sex groups, constituting the reference values. In the analysis by level of physical activity according to IPAQ-SF, median values of the number of repetitions of 33 for low and moderate, and 38 for the high level, the difference between groups was statistically significant ($p<0.001$) (Figure 2).

When comparing Strassmann's values with this research, the former is higher, obtaining an average of 41.3 vs. 34.9 respectively ($p<0.001$) (Supplementary material). In the linear regression of these data, an R^2 value of 0.226 ($p<0.001$) was obtained.

The predictive equations for men:

1minSTST Men = 43.394 - (age * 0.319) + (height * 0.029)

$R^2=0.346$

Predictive equations for women:

1minSTST Women = 90.583 - (age * 0.285) - (height * 0.272)

$R^2=0.26$

In the equation, age must be provided in years and height in centimeters.

Discussion

This study has established reference values to facilitate the comparative analysis of 1min-STS performance, aiming to enhance the interpretation of clinical outcomes. These values were derived from a diverse multicentric sample of individuals ranging from 18 to 80 years old in a developing country with similar characteristics to other Latin American populations. Including such a varied and representative population allows for applying these reference values in similar contexts where local normative data might be lacking. By offering these reference values, this research aims to provide valuable insights and guidance for

assessing the 1min-STST performance in populations with comparable demographic characteristics, thus aiding healthcare professionals and researchers in their endeavours to evaluate functional capacity in a more contextually relevant manner.

The importance of reference values adapting to the local population cannot be overstated [18]. When assessing various health parameters and functional capacity, using standardised values based on data from other populations may not accurately reflect the unique characteristics and diversities of the Latin American population.

Regarding the influence of physical activity on the number of repetitions, our results are consistent with previous reports [29, 30]. Our data indicate that individuals with a high level of physical activity perform more repetitions than those with low and moderate levels of physical activity. This is similar to findings reported by Gürses et al, where healthy subjects with a high level of physical activity according to IPAQ averaged 50.5 repetitions compared to 41.7 in the moderate physical activity group [29]. The result is also similar to what was shown in people with diseases [30].

An important aspect of these reference values is the inclusion of subjects with a BMI of 35. The rationale behind this choice stems from the fact that the Latin American population exhibits significant obesity rates, often surpassing 25% of the total population [20]. Consequently, from a clinical perspective, it is

anticipated that between two to three out of every 10 end-users of these tests will present with obesity. It is noteworthy that our approach aligns with previous studies, as exemplified by Furlanetto et al., who also employed a BMI upper limit of 40 [31].

Furthermore, applying standardised values from other populations might lead to misinterpretations and misdiagnoses, as individual variations may not be adequately accounted for[32]. Therefore, the ATS/ERS Statement recommend having reference values from the same people on which the procedures will be applied [33].

The results regarding whether a learning effect exists are contradictory. Although some articles suggest its [34] presence while others argue against it [26, 35], it's important that if a repetition is performed, strategies should be employed to minimize or nullify this learning effect. Several studies demonstrating no learning effect have actively demonstrated the technical movement to the subject or stopped the test immediately upon detecting errors in execution [36]. Undoubtedly, it is necessary to evaluate whether these strategies, as well as others that may emerge in the future, effectively eliminate this effect.

Another important aspect that lacks consensus is the height of the chair. The literature had reports chair heights between 43 and 48 cm [12, 37, 38]. In the Chilean context, chairs acquired for healthcare institutions do not have a standard size; however, they tend to range between 43-46 cm. For this reason, to facilitate the execution of the test and the applicability of reference values, especially in

primary healthcare settings, we chose this range. Undoubtedly, studies are needed to compare what the ideal chair height should be. However, we believe that more important than the chair height is the distance between the floor and the knee of the subjects to establish the ideal height.

An important point to consider is how representative the sample is within the context of the Chilean population. While Strassman et al.'s study included nearly 7,000 subjects [19], reaching such a number is quite challenging, especially in the over 60 age group. Comparatively, in studies from Latin America, Furlanetto et al. evaluated nearly 300 individuals in Brazil [31], a country with a population of 215 million. Our reference values were derived from just under 500 subjects in a population of 18 million. However, the sample size calculation was conducted with a confidence level of 97.5%, and to achieve a 95% confidence level, we only needed 386 subjects. While this may seem like an adequate number, like other reference value studies, it may be representative for adults up to 60 years old, but above that age, it becomes very difficult to find "healthy" older adult population.

Another point to highlight is that future research should explore what other variables influence the number of repetitions. Our data showed that age is the main factor, as did Strassman's data, however, this explained less than 30% of the test variance [19], suggesting that other parameters could improve the explanation of the 1min-STST variance. Many studies carried out in recent years in our population, especially in post-COVID-19 patients, have used Strassman

values [15, 39] . Since Swiss values are 30% higher than ours, there has been a subestimation of functional capacity. This reinforces the idea of having our own values for an adequate characterisation of the local population.

Our values are very similar to those shown by Furlanetto et al., who determined the reference values for 1min-STST in the Brazilian population [31]. For instance, the values are the same in women aged 60 to 69 (29 repetitions), which is also true for men aged 40 to 49 (31 repetitions). However, there is a significant difference with the values presented by Strassman et al. [19] For the same age ranges, Strassman's data show 33 repetitions in women aged 60 to 69, which is 10% higher than our data, and for men aged 40 to 49, Strassman's expected value is 45 repetitions, a 36% difference. This pattern is consistent and even more pronounced in younger age groups for both sexes, with Strassman's values surpassing our population's values by up to 12 repetitions.

Data previously reported by Strassman showed higher values in older male adults, however Furlanetto's values showed similar values [19, 31]. Our values differ from those shown in the literature since the male values are lower. The most likely explanation is possibly a selection bias due to a very small sample size in this subgroup.

One limitation of our data is the smaller number of subjects recruited over 60. This may be because older adults tend to have a sedentary lifestyle and a higher prevalence of non-communicable diseases, making it difficult to obtain a

population considered "healthy.". The categorisation of apparently healthy individuals was determined through self-reporting and the absence of diagnosed medical conditions. Nonetheless, it is worth noting that including individuals with undiagnosed conditions or diseases is a prevailing feature in studies involving extensive population samples aimed at establishing reference parameters. Another limitation of this study is that the sampling did not have a probabilistic approach. This could have introduced a bias in the selection of the participants since those who accepted the invitation could differ in characteristics from those who did not participate. An aspect not recorded was the socioeconomic characterisation that could have influenced the results. Finally, another limitation is that we perform only one repetition. The literature is not clear whether this test has a learning effect, however it seems that in healthy subjects or those without substantially reduced functional capacity there could be a learning effect that Furlanetto et al. estimated in one repetition [31].

Another recent article suggests three repetitions for the 1min-STS test is necessary to observe an individual's true performance in younger individuals, however in older adults only a single session may be sufficient [40]. There is undoubtedly no consensus on the number of tests required, and it appears that healthy individuals exhibit a higher learning effect than people with diseases. Therefore, future studies should explore if strategies such as practicing the execution of the test reduce this learning effect.

Conclusion

Reference values for the 1min-STST were determined for the healthy Chilean population aged 18 to 80 years. It provides a simple and inexpensive means of measuring functional capacity. These values can be valuable for assessing and determining the effects of interventions involving functional capacity evaluation through this test.

References

1. Barnekow-Bergkvist M, Hedberg G, Janlert U, Jansson E. Development of muscular endurance and strength from adolescence to adulthood and level of physical capacity in men and women at the age of 34 years. *Scand. J. Med. Sci. Sports* 1996; 6: 145–155.

2. Vanhees L, Lefevre J, Philippaerts R, Martens M, Huygens W, Troosters T, Beunen G. How to assess physical activity? How to assess physical fitness? *Eur. J. Cardiovasc. Prev. Rehabil.* 2005; 12: 102–114.

3. Liguori G. ACSM's guidelines for exercise testing and prescription. Lippincott Williams & Wilkins; 2020.

4. Torres-Castro R, Núñez-Cortés R, Larrateguy S, Alsina-Restoy X, Barberà JA, Gimeno-Santos E, García AR, Sibila O, Blanco I. Assessment of Exercise Capacity in Post-COVID-19 Patients: How Is the Appropriate Test Chosen? *Life* 2023; 13: 621.

5. Tran D. Cardiopulmonary Exercise Testing. *Methods Mol. Biol.* Methods Mol Biol; 2018; 1735: 285–295.

6. Singh SJ, Puhan MA, Andrianopoulos V, Hernandes NA, Mitchell KE, Hill

CJ, Lee AL, Camillo CA, Troosters T, Spruit MA, Carlin BW, Wanger J, Pepin V, Saey D, Pitta F, Kaminsky DA, McCormack MC, MacIntyre N, Culver BH, Sciurba FC, Revill SM, Delafosse V, Holland AE. An official systematic review of the European Respiratory Society/American Thoracic Society: measurement properties of field walking tests in chronic respiratory disease. *Eur. Respir. J.* 2014; 44: 1447–1478.

7. Fell BL, Hanekom S, Heine M. Six-minute walk test protocol variations in low-resource settings - A scoping review. *South African J. Physiother.* 2021; 77.

8. Peroy-Badal R, Sevillano-Castaño A, Torres-Castro R, García-Fernández P, Maté-Muñoz JL, Dumitrana C, Sánchez Rodriguez E, de Frutos Lobo MJ, Vilaró J. Comparison of different field tests to assess the physical capacity of post-COVID-19 patients. *Pulmonology* 2022; .

9. Kronberger C, Mousavi RA, Öztürk B, Dachs T-M, Rettl R, Camuz-Ligios L, Litschauer B, Badr-Eslam R. Exercise capacity assessed with the one-minute sit-to-stand test (1-min STST) and echocardiographic findings in patients with heart failure with preserved ejection fraction (HFpEF). *Heart Lung* 2022; 55: 134–139.

10. Spence JG, Brincks J, Løkke A, Neustrup L, Østergaard EB. One-minute sit-to-stand test as a quick functional test for people with COPD in general practice. *NPJ Prim. care Respir. Med.* 2023; 33: 11.

11. Tanriverdi A, Kahraman BO, Ozpelit E, Savci S. Test-Retest Reliability and Validity of 1-Minute Sit-to-Stand Test in Patients With Chronic Heart Failure. *Heart. Lung Circ.* Australia; 2023; 32: 518–524.

12. Bohannon RW, Crouch R. 1-Minute Sit-To-Stand Test: SYSTEMATIC REVIEW of PROCEDURES, PERFORMANCE, and CLINIMETRIC PROPERTIES. *J. Cardiopulm. Rehabil. Prev.* 2019; 39: 2–8.

13. Souza AC de, Alexandre NMC, Guirardello E de B. Psychometric properties in instruments evaluation of reliability and validity. *Epidemiol. e Serv. saude Rev. do Sist. Unico Saude do Bras.* 2017; 26: 649–659.

14. Sisó-Almirall A, Brito-Zerón P, Conangla Ferrín L, Kostov B, Moragas Moreno A, Mestres J, Sellarès J, Galindo G, Morera R, Basora J, Trilla A, Ramos-Casals M, Group OBOTCamfLC-S. Long Covid-19: Proposed Primary Care Clinical Guidelines for Diagnosis and Disease Management. *Int. J. Environ. Res. Public Health* 2021; 18: 4350.

15. Dalbosco-Salas M, Torres-Castro R, Rojas Leyton A, Morales Zapata F, Henríquez Salazar E, Espinoza Bastías G, Beltrán Díaz ME, Tapia Allers K, Mornhinweg Fonseca D, Vilaró J. Effectiveness of a Primary Care Telerehabilitation Program for Post-COVID-19 Patients: A Feasibility Study. *J. Clin. Med.* 2021; 10.

16. Holland AE, Malaguti C, Hoffman M, Lahham A, Burge AT, Dowman L, May AK, Bondarenko J, Graco M, Tikellis G, Lee JYT, Cox NS. Home-based or remote exercise testing in chronic respiratory disease, during the COVID-19 pandemic and beyond: A rapid review. *Chron. Respir. Dis.* 2020; 17: 147997312095241.

17. Dias JF, Oliveira VC, Borges PRT, Dutra FCMS, Mancini MC, Kirkwood RN, Resende RA, Sampaio RF. Effectiveness of exercises by telerehabilitation on pain, physical function and quality of life in people

with physical disabilities: a systematic review of randomised controlled trials with GRADE recommendations. *Br. J. Sports Med.* 2021; 55: 155–162.

18. Gräsbeck R. Reference values, why and how. *Scand. J. Clin. Lab. Invest. Suppl.* 1990; 201: 45–53.

19. Strassmann A, Steurer-Stey C, Lana KD, Zoller M, Turk AJ, Suter P, Puhan MA. Population-based reference values for the 1-min sit-to-stand test. *Int. J. Public Health* 2013; 58: 949–953.

20. Chávez-Velásquez M, Pedraza E, Montiel M. Prevalencia de obesidad: estudio sistemático de la evolución en 7 países de América Latina. *Rev. Chil. Salud Pública* 2019; 23: 72–78.

21. Leong DP, Teo KK, Rangarajan S, Kutty VR, Lanas F, Hui C, Quanyong X, Zhenzhen Q, Jinhua T, Noorhassim I, AlHabib KF, Moss SJ, Rosengren A, Akalin AA, Rahman O, Chifamba J, Orlandini A, Kumar R, Yeates K, Gupta R, Yusufali A, Dans A, Avezum Á, Lopez-Jaramillo P, Poirier P, Heidari H, Zatonska K, Iqbal R, Khatib R, Yusuf S. Reference ranges of handgrip strength from 125,462 healthy adults in 21 countries: a prospective urban rural epidemiologic (PURE) study. *J. Cachexia. Sarcopenia Muscle* Germany; 2016; 7: 535–546.

22. von Elm E, Altman DG, Egger M, Pocock SJ, Gøtzsche PC, Vandenbroucke JP. The Strengthening the Reporting of Observational Studies in Epidemiology (STROBE) statement: guidelines for reporting observational studies. *Lancet* Elsevier; 2007; 370: 1453–1457.

23. Lee PH, Macfarlane DJ, Lam TH, Stewart SM. Validity of the International

Physical Activity Questionnaire Short Form (IPAQ-SF): a systematic review. *Int. J. Behav. Nutr. Phys. Act.* 2011; 8: 115.

24. Ozalevli S, Ozden A, Itil O, Akkoclu A. Comparison of the Sit-to-Stand Test with 6 min walk test in patients with chronic obstructive pulmonary disease. *Respir. Med.* [Internet] 2006/06/27. England; 2007; 101: 286–293Available from: https://pubmed.ncbi.nlm.nih.gov/16806873.

25. Bohannon RW. Reference Values for the Five-Repetition Sit-to-Stand Test: A Descriptive Meta-Analysis of Data from Elders. *Percept. Mot. Skills* [Internet] SAGE Publications Inc; 2006; 103: 215–222Available from: https://doi.org/10.2466/pms.103.1.215-222.

26. Sevillano-Castaño A, Peroy-Badal R, Torres-Castro R, Gimeno-Santos E, García Fernández P, Garcia Vila C, Ariza Alfaro A, De Dios Álvarez R, Vilaró J, Blanco I. Is there a learning effect on 1-min sit-to-stand test in post-COVID-19 patients? *ERJ open Res.* 2022; 8.

27. Johnson MJ, Close L, Gillon SC, Molassiotis A, Lee PH, Farquhar MC, Bausewein C, Dorman S, Booth S, Oxberry S, Malik F, Simon S, Pattinson K, Yorke J, White P. Use of the modified Borg scale and numerical rating scale to measure chronic breathlessness: a pooled data analysis. *Eur. Respir. J.* 2016; 47: 1861–1864.

28. Cole TJ. The LMS method for constructing normalized growth standards. *Eur. J. Clin. Nutr.* 1990; 44: 45–60.

29. GÜRSES HN, DENİZOĞLU KÜLLİ H, DURGUT E, ZEREN M. Effect of Gender and Physical Activity Level on Sit-to-Stand Test Performance Among Young Adults. *Genç Yetişkinlerde Cinsiyet ve Fiz. Akt. Seviye.*

Otur-kalk Test Performansı Üzerine Etkisi. [Internet] 2020; 8: 222–226Available from: http://10.0.55.155/bas.galenos.2019.3541.

30. Domingues VL, Pompeu JE, de Freitas TB, Polese J, Torriani-Pasin C. Physical activity level is associated with gait performance and five times sit-to-stand in Parkinson's disease individuals. *Acta Neurol. Belg.* Italy; 2022; 122: 191–196.

31. Furlanetto KC, Correia NS, Mesquita R, Morita AA, do Amaral DP, Mont'Alverne DGB, Pereira DM, Pitta F, Dal Corso S. Reference Values for 7 Different Protocols of Simple Functional Tests: A Multicenter Study. *Arch. Phys. Med. Rehabil.* 2022; 103: 20-28.e5.

32. Lippi G, Blanckaert N, Bonini P, Green S, Kitchen S, Palicka V, Vassault AJ, Mattiuzzi C, Plebani M. Causes, consequences, detection, and prevention of identification errors in laboratory diagnostics. *Clin. Chem. Lab. Med.* 2009; 47: 143–153.

33. Miller MR, Crapo R, Hankinson J, Brusasco V, Burgos F, Casaburi R, Coates A, Enright P, van der Grinten CPM, Gustafsson P, Jensen R, Johnson DC, MacIntyre N, McKay R, Navajas D, Pedersen OF, Pellegrino R, Viegi G, Wanger J. General considerations for lung function testing. *Eur. Respir. J.* 2005; 26: 153–161.

34. Radtke T, Puhan MA, Hebestreit H, Kriemler S. The 1-min sit-to-stand test—A simple functional capacity test in cystic fibrosis? *J. Cyst. Fibros.* [Internet] 2016; 15: 223–226Available from: https://www.sciencedirect.com/science/article/pii/S1569199315002027.

35. Reychler G, Boucard E, Peran L, Pichon R, Le Ber-Moy C, Ouksel H,

Liistro G, Chambellan A, Beaumont M. One minute sit-to-stand test is an alternative to 6MWT to measure functional exercise performance in COPD patients. *Clin. Respir. J.* [Internet] 2017/06/15. England; 2018; 12: 1247–1256Available from: https://pubmed.ncbi.nlm.nih.gov/28621019.

36. Hardy S, Berardis S, Aubriot A-S, Reychler G, Gohy S. One-minute sit-to-stand test is practical to assess and follow the muscle weakness in cystic fibrosis. *Respir. Res.* England; 2022; 23: 266.

37. Vilarinho R, Montes AM, Noites A, Silva F, Melo C. Reference values for the 1-minute sit-to-stand and 5 times sit-to-stand tests to assess functional capacity: a cross-sectional study. *Physiotherapy* [Internet] 2024; Available from: https://www.sciencedirect.com/science/article/pii/S0031940624000063.

38. Kuo Y-L. The influence of chair seat height on the performance of community-dwelling older adults' 30-second chair stand test. *Aging Clin. Exp. Res.* [Internet] 2013; 25: 305–309Available from: https://doi.org/10.1007/s40520-013-0041-x.

39. Núñez-Cortés R, Rivera-Lillo G, Arias-Campoverde M, Soto-García D, García-Palomera R, Torres-Castro R. Use of sit-to-stand test to assess the physical capacity and exertional desaturation in patients post COVID-19: *Chron. Respir. Dis.* SAGE PublicationsSage UK: London, England; 2021; 18: 1479973121999205.

40. Vilarinho R, Montes AM, Melo C. Age-related influence on reliability and learning effect in the assessment of lower limb strength using sit-to-stand tests: A cross-sectional study. *Heal. Sci. Reports* [Internet] John Wiley &

Sons, Ltd; 2024; 7: e2064Available from: https://doi.org/10.1002/hsr2.2064.

Variable	All (n=499)	Women (n=287)	Men (n=212)
N by age group (%)			
- 18-29	132 (26.5)	67 (23.3)	65 (30.7)
- 30-39	70 (14.0)	35 (12.2)	35 (16.5)
- 40-49	89 (17.8)	61 (21.3)	28 (13.2)
- 50-59	67 (13.4)	43 (15)	24 (11.3)
- 60-69	72 (14.4)	45 (15.7)	27 (12.7)
- 70-80	69 (8.2)	36 (12.5)	33 (15.6)
Height(m), median (IR)	1.63 (0.14)	1.58 (0.10)	1.70 (0.10)
Weight (Kg), median (IR)	72.8 (20)	67.5 (18)	80 (17.6)
BMI (kg/m^2), mean (SD)	27.3 ± 4.1	27.3 ± 4.2	27.2 ± 3.9
Physical activity level (IPAQ-SF)			
Low (%)	360 (72.1)	216 (75.3)	144 (67.9)
Moderate (%)	91 (18.2)	51 (17.8)	40 (18.9)
High (%)	48 (9.6)	20 (7.0)	28 (13.2)
Tobacco use			
Smoker (%)	126 (25.3)	81 (28.2)	45 (21.2)
Non smoker (%)	349 (69.9)	192 (66.9)	157 (74.1)
Former smoker (%)	24 (4.8)	14 (4.9)	10 (4.7)
Leg Fatigue pre 1min-STST (Borg scale), median (IR)	0 (1)	0 (1)	0 (1)
Leg Fatigue post 1min-STST (Borg scale), median (IR)	3 (3)	4 (2)	3 (3)

Table 1: Characteristics of the Study Population. The data have been presented as mean (standard deviation), median (interquartile range), or frequency (percentage), as appropriate. BMI. Body Mass Index; IPAQ-SF: International Physical Activity Questionnaire - Short Form. 1min-STST: One-minute sit-to-stand test.

Age group (years)	Number of STS repetitions									
	Women					Men				
	p2.5	p25	p50	p75	p97.5	p2.5	p25	p50	P75	p97.5
18-29	28	33	38	45	61	27	32	38	47	61
30-39	23	33	38	44	60	26	31	39	47	55
40-49	22	28	33	38	47	26	30	30	37	58
50-59	20	26	32	37	53	20	28	32	39	58
60-69	17	23	28	33	49	15	23	25	30	37
70-80	17	21	24	30	47	15	20	23	26	31

Table 2: The percentiles are presented according to sex and age categories, along with the delineation of cut-off values and the value denoting normality. Additionally, the Lower Limit of Normal (LLN) at the 2.5th percentile and the Upper Limit of Normal (ULN) at the 97.5th percentile are defined. With the LLN value, we will be able to determine which individuals, according to sex and age, are below the normal range. The p50 corresponds to the median, and the p25 and p75 correspond to Q1 and Q3, respectively.

Figure legends

Figure 1: Association between the number of repetitions of the 1min-STST and age in men and women. N° of rep: Number of repetitions.

Figure 2: Comparison of 1min-STST by physical activity level

Click here to access/download;Figure;Figure 1 July 2.pdf

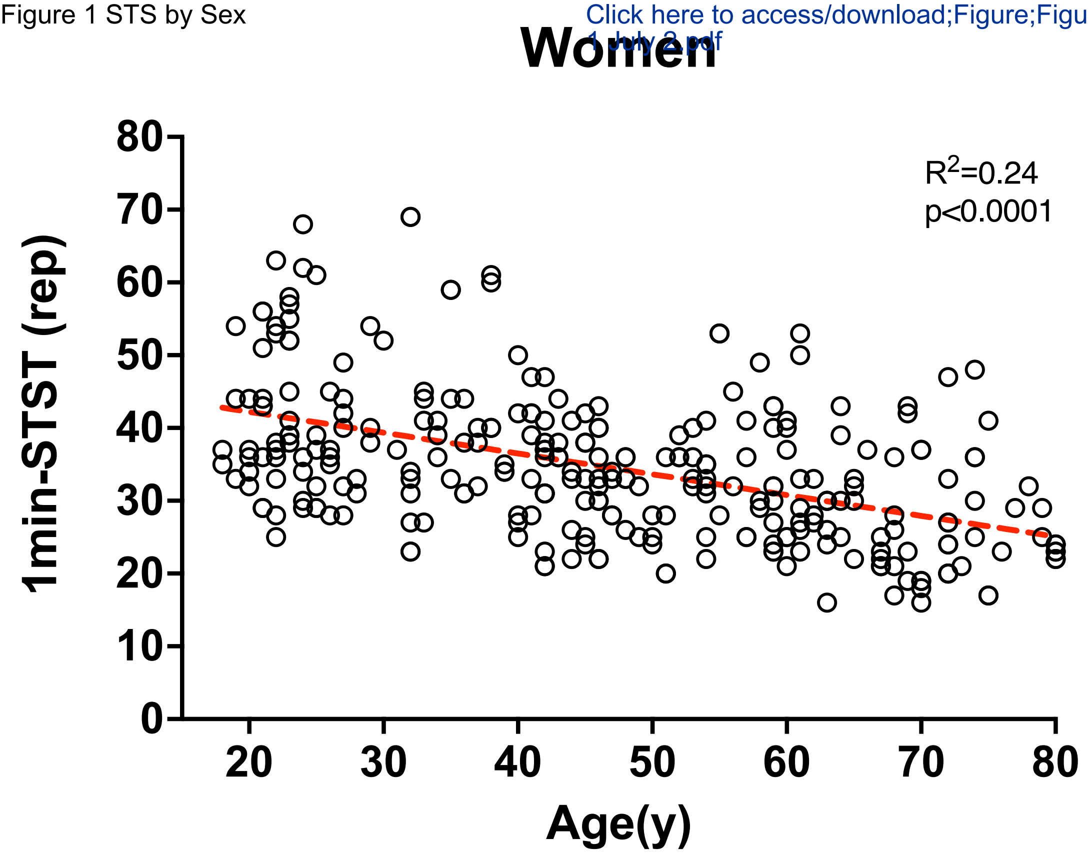

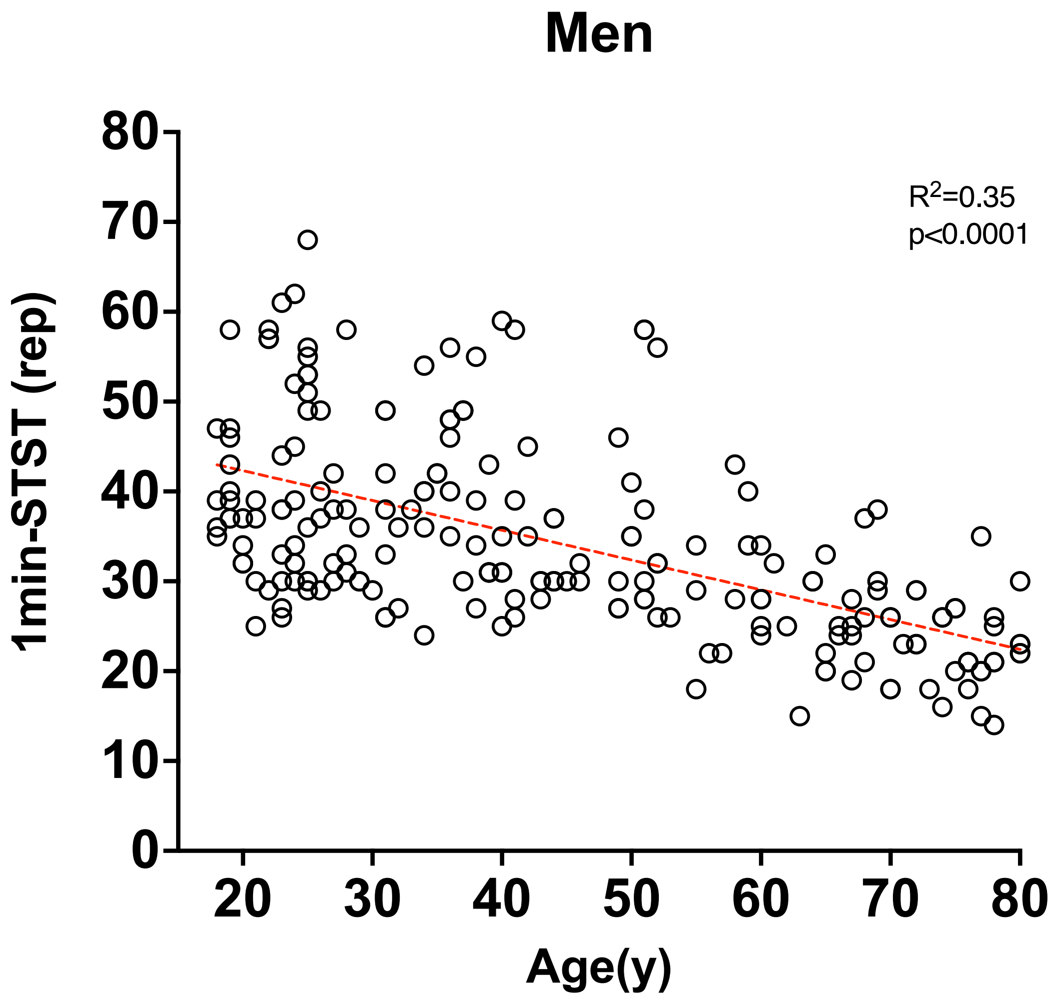

Figure 2 STS and IPAQ

Click here to access/download;Figure;Fig

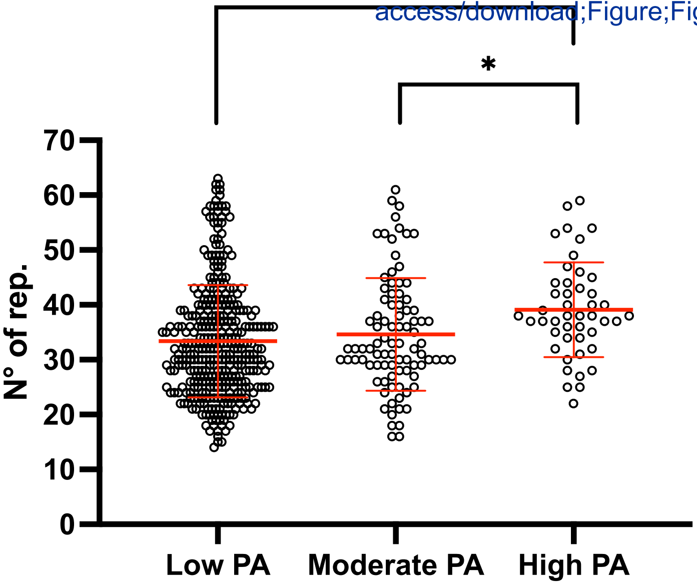

Supplement: Attachment — Submitted filename: PONE-D-24-27131.pdf [file pone.0317594.s002.pdf]
