# Supplementary material for: One-minute sit-to-stand test: Reference values for the Chilean population
Source: PLoS One. 2025 Jan 29;20(1):e0317594. doi: 10.1371/journal.pone.0317594 (PMC11778653; doi:10.1371/journal.pone.0317594)
Supplement: Attachment — Submitted filename: Response to Reviewers.docx [file pone.0317594.s003.docx]

December 6, 2024

Dear Editor,

The revised version of the manuscript entitled “One-minute sit-to-stand test: Reference values for Chilean population” (PONE-D-24-27131)” is currently being submitted to the Journal for consideration by the editor and reviewers.

First, we would like to express our gratitude for the opportunity of submitting the current revised version of our manuscript. The material has been modified following your recommendations. At the end of this document, you will find the point-by-point responses to the reviewers. For a better location of the corrections, we highlighted with “track changes” the modified text in the manuscript.

Finally, we appreciate the reviewers' comments that improved the revised manuscript's quality. We hope that the revised version of our manuscript has met the reviewer's and the editor´s expectations.

Best regards,

Rodrigo Torres Castro

Rebuttal

| Reviewer 1 | Authors could specify their conclusion and clinical importance | Thank you very much for the suggestion. The conclusion has been modified to provide further detail as requested. |
| --- | --- | --- |
|  | Introduction: -It is too long, you can try to combine some sentences like line 77-79 | Thank you very much for the observation. Some parts of the introduction were merged to reduce its length. |
|  | -Please explain the "specific health conditions" in line 126-127 | Thank you for pointing this out. We have revised the sentence to specify the factors directly, such as chronic diseases, medication use, and particular physiological states (e.g., pregnancy, fasting, or posture), which can significantly influence test results. As highlighted by Grasbeck (reference 18), reference values should take into account individual-specific factors to ensure comparability between observed values and reference values. |
|  | Methods: - Line 168: You can change it to IPAQ-SF, because you used this term later without abbreviation. | Thank you very much for the correction. The acronym has been changed to IPAQ-SF. |
|  | - Line 220: Why did you choose %97 for confidence level? | We chose a confidence level of 97% to increase the precision of our population parameter estimates. This decision aimed to reduce uncertainty and obtain a narrower confidence interval. Given the nature of our study, minimizing the risk of misinterpretation of results was particularly important, which justified using a confidence level slightly above the conventional 95%. |
|  | - Line 179: Please correct “[26]..” | Thank you. The error has been corrected. |
|  | -How did you organize the separation of the participants to the age groups? | Thank you for the suggestion. The participants were categorized into the following age groups: 18-29, 30-39, 40-49, 50-59, 60-69, and 70-80 years. We have added this information to the methods section |
|  | Results: - Use “moderate” istead of moderated | Thank you for the correction. It has been revised to 'moderate'. |
|  | Discussion: - Consider adding more detail about learning effect | Thank you very much for the suggestion. The paragraph has been restructured to place greater emphasis on the learning effect. |
|  | - Which results Socioeconomic characterisation can influence? | Thank you for pointing this out. We acknowledge that the socioeconomic status of participants was not recorded, which could potentially influence the results of the 1min-STST performance. As demonstrated by Shishehbor et al. (2006), lower socioeconomic status is associated with reduced functional capacity, possibly due to limited access to healthcare, nutrition, and opportunities for physical activity. These factors may impact test outcomes, making socioeconomic characterization relevant to better interpret the findings. This limitation has been recognized in the discussion, and we suggest that future studies incorporate socioeconomic characterization to understand its impact on performance outcomes more comprehensively.  Shishehbor, M. H., Litaker, D., Pothier, C. E., & Lauer, M. S. (2006). Association of socioeconomic status with functional capacity, heart rate recovery, and all-cause mortality. *Jama*, *295*(7), 784-792. |
| Reviewer 2 | Some errors were found in the English writing, so I suggest that the article be sent for review before final submission. | Thank you very much for the suggestion. The manuscript has been sent to a scientific writing editing company for review |
|  | The inclusion criteria include adults between 18 and 80 who self-reported as healthy. How do you evaluate healthy considering that 150 of the 499 participants are smokers or former smokers? Do you think that this could have biased your results? | Thank you for raising your concern regarding the inclusion criteria and the classification of participants as 'healthy.' In this study, we applied the Royal College of Physicians' definition of 'healthy,' which considers individuals without significant diseases or functional limitations that could affect their performance in the test to be healthy. Consequently, although some of our participants were smokers or former smokers, they were included as long as they did not present active conditions that could influence their performance. This inclusion also allowed us to better reflect the diversity of the Chilean population, where approximately 30% of adults identify as smokers. Furthermore, we conducted an exploratory comparison between smokers, former smokers, and non-smokers, and found no significant differences in the number of repetitions in the 1min-STST. We have added this information to the Methods section to clarify our approach to classifying participants as healthy. |
